# Device for measuring bronchodilator delivery and response in resource-limited settings

**DOI:** 10.1186/cc12103

**Published:** 2013-03-19

**Authors:** CW Carspecken, D Talmor

**Affiliations:** 1 Harvard Medical School and Beth Israel Deaconess Medical Center, Boston, MA, USA

## Introduction

Effective delivery of aerosolized bronchodilators for patients with asthma is crucial for adequate therapy in critical care and emergent settings. Often administered with pressure-metered dose inhalers (pMDIs), bronchodilator delivery depends on the correct patient technique during administration [1] and the ability to measure treatment response, which are difficult to monitor at the point of care and particularly so in resource-poor settings where standard in-hospital monitoring is unavailable [2].

## Methods

**Figure 1:**
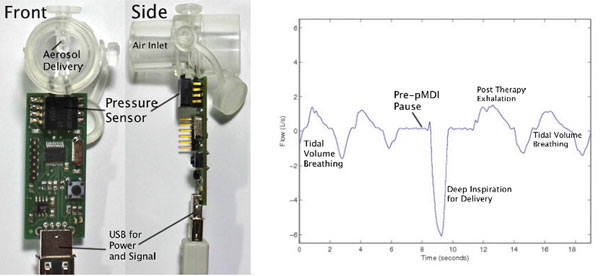


A point-of-care device for airflow measurement during bronchodilator delivery was designed and tested for use in resourcelimited settings. The handheld device was constructed from a clinical aerosol delivery tube with a bidirectional sensor for pressure differential detection about the aerosol element (Figure 1). The custom low-cost (<$11) electronics were designed such that no power supply would be needed apart from a computer or mobile device. Collection of airflow signal and calibration was performed with a standard 3 l syringe with flow volume measurement on one adult subject.

## Results

Calibration of the assembled device over range of 0.5 to 3 l/ second enabled conversion of pressure difference to air flow with a mean measured tube resistance of 0.258 (kPa*second)/l. Robust signal responses to an adult subject's continuous respiratory maneuvers on the tube itself were demonstrated. Subject performance of the pMDI technique with subsequent tidal volume breathing was recorded and analyzed (Figure 1).

## Conclusion

Design and calibration of a novel low-cost monitoring device for bronchodilator delivery monitoring enabled detection and recording of characteristic flow volume respiratory patterns for point-of-care diagnostics in resource-limited settings. Future work will require clinical testing and automated detection of the correct pMDI patient technique.
